# The impact of the catheter to vein ratio on peripheral intravenous cannulation success, a post-hoc analyses

**DOI:** 10.1371/journal.pone.0252166

**Published:** 2021-05-24

**Authors:** Fredericus H. J. van Loon, Hendrikus H. M. Korsten, Angelique T. M. Dierick–van Daele, Arthur R. A. Bouwman

**Affiliations:** 1 Department of Anesthesiology, Intensive Care and Pain Medicine, Catharina Hospital, Eindhoven, The Netherlands; 2 Department of Science and Technology in Anesthesia Nursing Practice, Fontys University of Applied Sciences, Eindhoven, The Netherlands; 3 Department of Signal Processing Systems and Electrical Engineering, TU/e University of Technology, Eindhoven, The Netherlands; 4 Department of People and Health Sciences, Fontys University of Applied Sciences, Eindhoven, The Netherlands; 5 Department of Research and Education, Catharina Hospital, Eindhoven, The Netherlands; 6 Department of Anesthesiology, Intensive Care and Pain Medicine, Catharina Hospital, Eindhoven, The Netherlands; Ohio State University Wexner Medical Center Department of Surgery, UNITED STATES

## Abstract

**Background:**

Intravenous cannulation is usually the first procedure performed in modern healthcare, although establishing peripheral intravenous access is challenging in some patients. The impact of the ratio between venous diameter and the size of the inserted catheter (catheter to vein ratio, CVR) on the first attempt success rate can be of added value in clinical. This study tries to give insight into the consideration that must be made when selecting the target vein and the type of catheter, and proved the null hypothesis that an optimal CVR would not be associated with increased first attempt cannulation success.

**Methods:**

This was a post-hoc analyses on adult patients admitted for peripheral intravenous cannulation. Intravenous cannulation was performed according to practice guidelines, by applying the traditional landmark approach. The CVR was calculated afterwards for each individual patient by dividing the external diameter of the inserted catheter by the diameter of the target vein, which was multiplied by 100%.

**Results:**

In total, 610 patients were included. The median CVR was 0.39 (0.15) in patients with a successful first attempt, whereas patients with an unsuccessful first attempt had a median CVR of 0.55 (0.20) (*P*<0.001). The optimal cut-off point of the CVR was 0.41. First attempt cannulation was successful in 92% of patients with a CVR<0.41, whereas as those with a CVR>0.41 had a first attempt success rate of 65% (*P*<0.001).

**Conclusion:**

This first introduction of the CVR in relation to cannulation success should be further investigated. Although, measuring the venous diameter or detection of a vein with a specific diameter prior to cannulation may increase first attempt cannulation success.

## Introduction

Intravenous cannulation is usually the first procedure performed by anesthesia providers on patients presenting for procedures requiring anesthesia or procedural sedation [1]. Anyway, peripheral intravenous cannulation is a common clinical procedure in today’s healthcare setting, although establishing peripheral intravenous access is challenging in some patients [2, 3]. These patients endure multiple failed attempts to obtain vascular access. Several risk factors for failed peripheral intravenous cannulation were identified, which was topic of many recent publications. These risk factors mostly include patient related factors, of which palpability and visibility of the vein are frequently mentioned factors [4]. Notwithstanding, if there is an impossibility to detect a suitable vein by palpating and visualizing the extremity, additional technologies like ultrasound should be used to identify a target vein.

The size of the inserted intravenous catheter depends on the clinical situation, due to the fact that larger sized catheters ensure faster administration of fluids [1]. Though, for the insertion of larger sized catheters, larger sized veins should be selected. Peripheral veins with a smaller diameter are an important risk factor for failed intravenous cannulation [4]. The same applies to the insertion of smaller sized peripheral intravenous catheters, according to recent publications [5]. Witting et al. previous described that an venous cross-sectional area of more than 4 squared centimeters results in increased first attempt cannulation success [6].

The risk for failed cannulation on the first attempt can be predicted with the A-DIVA scale [4]. A higher score on this five-variable scale indicates the likelihood for failed cannulation, based on the presence of a difficult intravenous access [4]. One of the factors included in this scale is the venous diameter [4]. A venous diameter less than 3 millimeters is an important risk factor for failed cannulation on the first attempt [4]. Logically, venous diameter correlates significant to the venous cross-sectional area, as shown in a previous study [7]. Therefore, a venous diameter of 2 millimeters results in a cross-sectional area of 3.14 squared millimeters, whereas a venous diameter of 3 millimeters results in a cross-sectional area of 7.07 squared millimeters. In line with the results as published by Witting et al., it is assumed that the cut-off value for venous cross-sectional area of 4 squared millimeters is decisive for cannulation success [6].

Actually, in our believe, cannulation site should be chosen based on the combination of the venous diameter and the size of the intended catheter. The impact of the ratio between these two factors on the first attempt success rate was, to the best of our knowledge, never studied before. Nonetheless, it seems trivial that the venous diameter must match the size of inserted catheter to guarantee cannulation success. It can be of added value to know the catheter to vein ratio (CVR) of the individual patient admitted for intravenous cannulation. To add on this, particularly patients with an increased risk for difficult intravenous cannulation as a result of smaller peripheral veins will benefit from the calculation of the CVR prior to catheter insertion [4].

The coherence between venous diameter and the size of the inserted catheter on the first attempt success rate of cannulation was the outcome of interest in this study. Moreover, this study tries to give the clinician insight into the consideration that must be made when selecting the target vein and the type of catheter. Therefore, the current study proved the hypothesis that an optimal CVR coherent with increased first attempt cannulation success. The null hypothesis of the current study is that an optimal CVR would not be associated with increased first attempt cannulation success.

## Materials and methods

### Design and setting

The current study is based on a previous dataset and was set up as post-hoc analyses [4]. Data was initially collected between January and May 2018. For the analyses in the current study, the dataset was assessed between October and December 2020 [4]. In the initial study, focus was on the identification of risk factors for difficult intravenous cannulation [4]. The studied cohort included surgical patients who were recruited from the holding area of the theatre complex (Catharina Hospital, Eindhoven, The Netherlands) [4]. Data were collected by the depending anesthesiologist or nurse anesthetist during the procedure of intravenous cannulation by asking the patient or from the preoperative anesthesia chart, and registered on score forms [4]. Completed score forms were included in the dataset and analyzed [4]. The institutional review board (Catharina Hospital, Eindhoven, The Netherlands) approved the study protocol (ref: 2015–21) and gave permission to use collected data for secondary analyses [4]. Written informed consent was obtained from all patients.

### Participants

Data regarding peripheral intravenous cannulation in 610 patients were recorded, including venous diameter and catheter size. Patients included adults, who were asked for participation regardless their American Society of Anesthesiology (ASA) physical status, demographics and medical history. Those patients who did not understand or answer the questionnaire (due to physical or communicational disorders), were unresponsive, or already had a peripheral intravenous cannulation inserted were excluded. Patients received usual care throughout the study. No a priori sample size calculation was performed, because this was an observational study. Patients were randomly recruited from the initial dataset [4]. Collected data was anonymized and therefore not traceable to the individual patient.

### Procedure

The performed procedure of peripheral intravenous cannulation was according to the previous publication by Van Loon et al [4]. A short peripheral intravenous catheter was inserted in the upper extremity, including the cephalic, basilic and median veins [8]. Venous dilation was created after the application of a tourniquet, surrounded on the upper extremity five centimeters proximal to the puncture site for at least one minute. Peripheral intravenous cannulation was performed prior to induction of anesthesia or procedural sedation. Practitioners, both anesthesiologists and nurse anesthetists, with a minimum of one year of experience in peripheral intravenous cannulation, performed the procedures and measurements in this study. Intravenous cannulation was performed according to practice guidelines, by applying the traditional landmark approach of palpating and visualizing the extremity [8, 9]. The size of the inserted intravenous catheters ranged from 14 to 22 gauge and was chosen by the depending practitioner based on the clinical situation [8, 9]. Catheters from a single manufacturer were used throughout the study, which were routinely used in the hospital (Venflon Pro Safety; BD Infusion Therapy AB, Helsingborg, Sweden). All cannulations were performed in the same environment, with a constant temperature of 19 ±2 degrees Celsius.

### Measurements

The outcome of interest was defined as successful peripheral intravenous cannulation on the first attempt [4]. Intravenous cannulation was considered successful if the practitioner was able to inject a saline flush without signs of infiltration [9]. The CVR was calculated for each individual patient by dividing the external diameter of the inserted catheter by the diameter of the target vein [10, 11]. Any relation between first attempt cannulation success and a patients CVR was sought during analyzing the data. The venous diameter was measured by placing a ruler on the extremity of the patient. This measurement was performed prior to cannulation, one minute after the application of a tourniquet on a participants upper extremity. Measurements were performed by the practitioner performing the procedure of peripheral intravenous cannulation. All of these were trained in optimal performing measurements in a briefing prior to the start of the study. A practical and accessible technique was chosen, wherefore no ultrasound was used to measure venous size. Measurements were registered on for this study designed forms, of which the collected data were afterwards entered in a SPSS (version 27, SPSS Inc., Chicago, Illinois, USA) data matrix.

### Statistical analyses

Descriptive statistics were used to analyze data related to the studied population. These were represented as mean and standard deviation or median and interquartile range, based on a normal distribution, or as proportions with percentages. Venous diameter was represented as both continues variables and nominally between 1 and 6 millimeters with steps of 1 millimeter. To detect differences between study groups, Mann-Whitney U and Kruskal Wallis H testing was used as appropriate. A receivers operating curve (ROC) and area under the curve (AUC) was plotted to determine the optimal cut-off point based on a maximum 100-sensitivity and specificity [12]. Spearman’s ρ was calculated to detect a correlation between variables. Throughout the analyses, *P*<0.05 was considered as statistically significant. SPSS was used for the analysis.

## Results

Patients demographics are represented in Table 1. All patients were in stable hemodynamic condition.

**Table 1:** Demographics of the included patients.

Variable	*Description*	Value
Sex	*Male*	342 (56%)
	*Female*	268 (44%)
Age		55 ±14
BMI		28 ±5
Number of attempts to successful cannulation	*1 attempt*	506 (83%)
	*2 attempts*	67 (11%)
	*3 attempts*	19 (3%)
	*4 attempts*	18 (3%)

A median CVR of 0.41 (0.20) was registered throughout the studied population. For patients with a successful first attempt of peripheral intravenous cannulation, the median CVR was 0.39 (0.15), whereas patients with an unsuccessful first attempt had a median CVR of 0.55 (0.20) (*P*<0.001, U = 43566, Z = -9.66). A significant correlation between a patients CVR and first attempt cannulation success was obtained (*P*<0.001, ρ = 0.297). Calculations of the CVR regarding the different sizes of inserted catheter are as represented in Table 2, which differed statistically (*P*<0.001, H = 47, df = 4). In general, venous diameter was smaller in patients with a failed first attempt (2.3 ±1.1 millimeters) when compared to those with a successful first attempt of cannulation (3.2 ±1.1 millimeters), with *P*<0.001 (U = 41991, Z = -10.53). In addition, the likelihood for a failed first attempt of intravenous cannulation increased as the CVR increased, due to a smaller venous diameter.

**Table 2:** Relevant outcomes regarding the different study groups, based on the size of the inserted catheter.

Size of the inserted catheter	Success rate on the first attempt	Catheter to vein ratio	Venous diameter
22 gauge	50 (66%)	0.50 (0.33)	2.0 (0.9)
20 gauge	113 (77%)	0.46 (0.27)	2.6 (1.0)
18 gauge	208 (90%)	0.38 (0.18)	3.4 (1.0)
16 gauge	76 (89%)	0.40 (0.23)	4.3 (1.2)
14 gauge	61 (86%)	0.43 (0.14)	4.8 (0.7)

Success rate on the first attempt is represented as proportions (percentages). Catheter to vein ratio and venous diameter in millimeters are represented as median (interquartile range).

Regarding the venous diameter, the CVR decreased as the diameter of the vein increased. In veins with a diameter of 1 millimeter, the median CVR was 0.98. Additionally, veins with a diameter of 2, 3, 4, 5 or 6 millimeters had a median CVR of respectively 0.52, 0.38, 0.30, 0.28 and 0.22.

**Fig 1:**
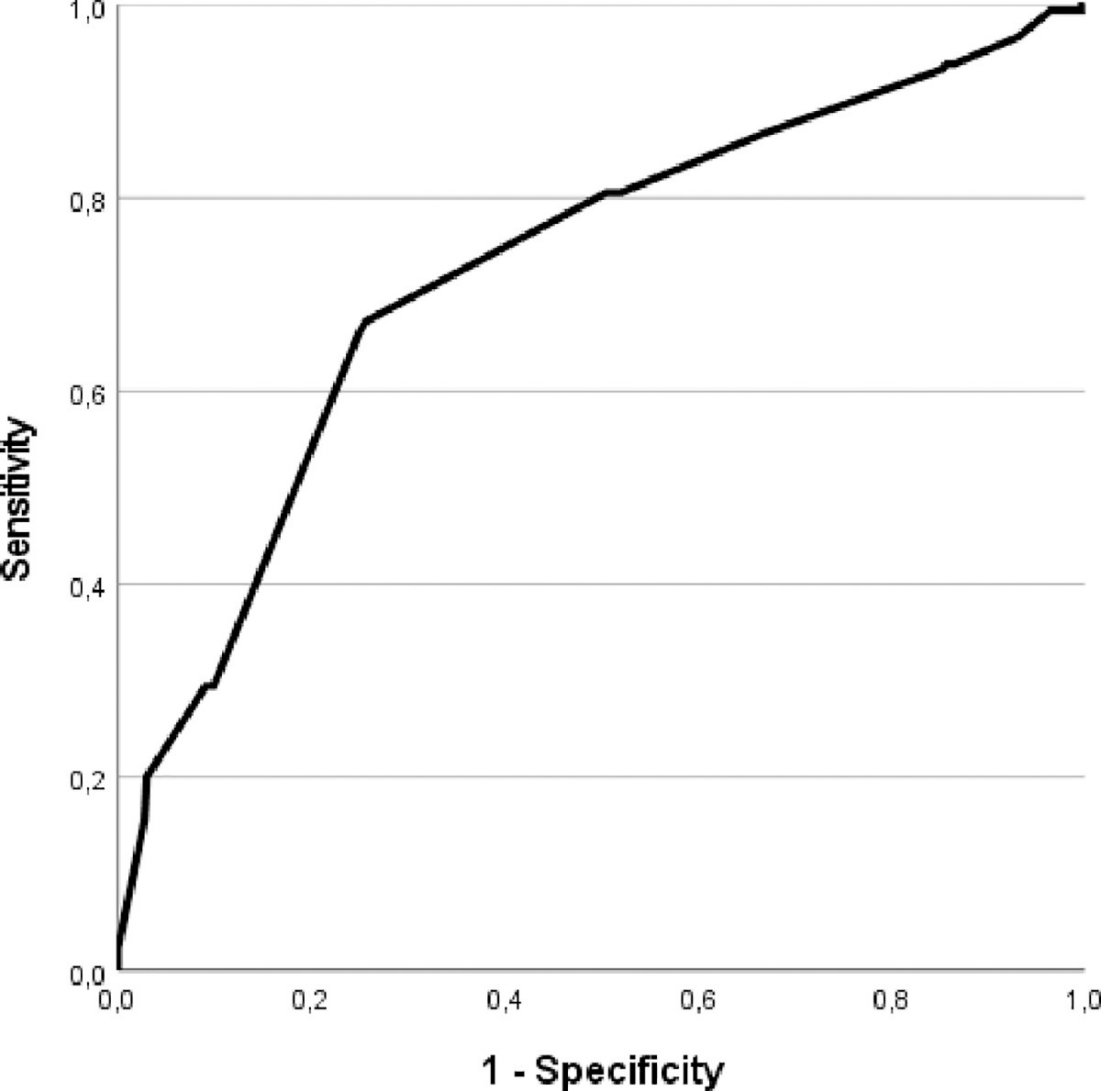
ROC curve to determine the optimal cut-off point of the CVR. ROC curve regarding the CVR, based on the outcome of interest, with an AUC of 73% (68% to 77%) and optimal cut-off point of 0.41 (100-specificity 0.26 and sensitivity 0.67).

**Fig 2:**
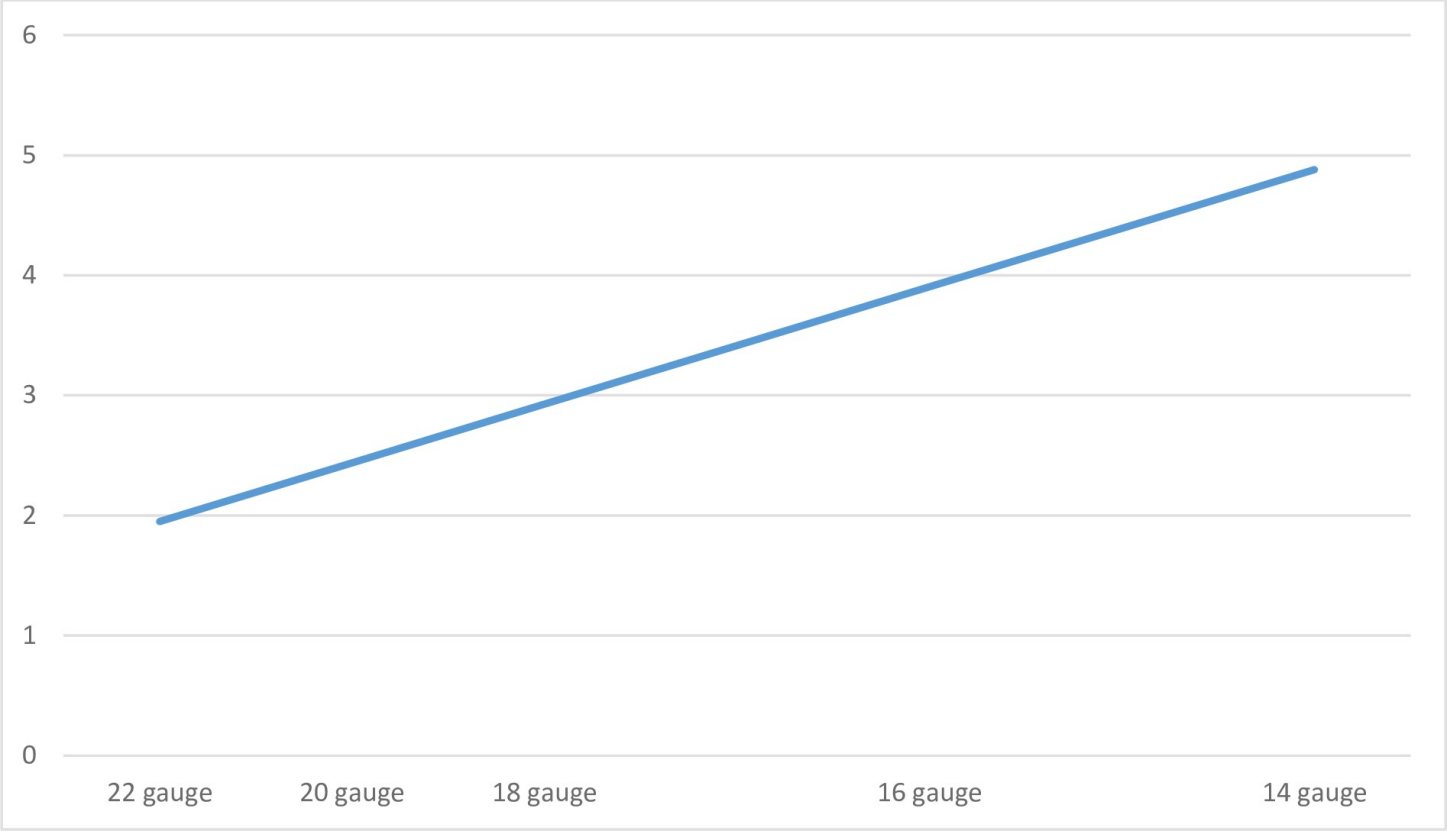
The relation between venous diameter and the size of the inserted catheter to guarantee a CVR<41%. Venous diameter in millimeters (vertical axes) and the size of the inserted catheter (horizontal axes).

ROC analysis was undertaken comparing 100-sensitivity to specificity for CVR on the outcome of interest (Fig 1), with an AUC of 73% (68% to 77%). The optimal cut-off point of the CVR on this curve was 0.41 (100-specificity 26% and sensitivity 67%). Based on this, first attempt cannulation was successful in 92% of patients with a CVR<0.41, whereas those with a CVR>0.41 had a first attempt success rate of 65%, resulting in a relative risk of 0.24 (0.18 to 0.31) (*P*<0.001, χ^2^ = 117.35, df = 1). Fig 2 represents the relation between the minimum venous diameter that is needed for the type of catheter to guarantee a CVR<0.41.

## Discussion

To our knowledge, this is the first study identifying the relation between first attempt cannulation success of peripheral veins with the calculated CVR of the individual patient. As a result of this study, the chance of successful cannulation on the first attempt is increased in patients with a CVR up to 0.41. A CVR greater than 0.41 resulted in an increased risk for failed cannulation on the first attempt. Briefly, a patients CVR correlated significantly with first attempt cannulation success.

The CVR was previously investigated in relation to the risk for symptomatic venous thromboembolism, especially in patients with a peripherally inserted central catheter (PICC) [10, 13]. Calculating a patients CVR can guide clinicians in selecting a vascular access device. Device selection is normally based on the clinical situation in clinical practice, as supposed by an expert panel from the United Kingdom (UK) [14]. The Intravenous Nurses Society (INS) guideline, on the contrary, stated to select the smallest and shortest sized catheter to accommodate the intended therapy [15]. Strauss et al. reported that the majority of answers given by clinicians on the question ‘which catheter did you choose and why?’ reveals confusion and a disturbing reliance on habit and tradition as well as on factors such as availability and procurement habits of their institution [1].

The purpose of the previously created A-DIVA scale was to determine a patients a priori risk for difficult intravenous cannulation, of which a smaller sized vein is one of the factors on that scale [4]. The current study was set up as a post-hoc analysis, with focus on the diameter of the target vein. Some patients simply suffer from smaller sized veins, but need a peripheral intravenous catheter for different indications. Calculation of the CVR, on that account, can guide catheter selection, and is therefore suggested to increase cannulation success. The results of the current study can therefore be seen as an addition to the A-DIVA scale and are particularly relevant for those patients who score positive for the presence of a venous diameter smaller than three millimeters on the A-DIVA scale [4].

Larger sized devices are indicated for surgical and trauma patients, or those admitted for blood transfusions, rapid infusion of large volumes or viscous liquids [1]. On the other hand, smaller sized catheter are used in fragile-veined patients like children and elderly, or for general and intermittent infusions [1]. Regarding the current study results, while considering the cut-off CVR of 0.41 to increase cannulation success, the venous diameter should be at least 1.95 millimeters for the insertion of a 22-gauged catheter. In addition, for each increase of 1 millimeter in the external diameter of the catheter, venous size should be increased with 2.44 millimeters. Hence, when a clinician is planning to insert a 14-gauged device, for instance, venous diameter should be at least 4.88 millimeters to guarantee a CVR of 0.41.

Logically, cannulation of smaller sized veins lead to an increased risk for failed attempts [4]. Moreover, a venous diameter of at least 2 millimeters is a risk factor for failed cannulation according to the A-DIVA scale [4, 16]. In line with this, smaller sized veins are associated with a lower CVR. The cut-off CVR of 0.41 was calculated with the Youden’s statistic, based on the 100-specificity and sensitivity. The AUC of the ROC was 73% and is therefore denoted to be generally acceptable [12]. Though, an AUC greater than 80% is stated as strongly acceptable regarding its discriminability [12, 17]. Both smaller sized veins and larger sized catheters are associated with an increased CVR, with an increased risk for failed attempts of intravenous cannulation. On the contrary, a CVR smaller than 0.41 can be achieved by selecting larger sized veins or smaller sized catheters.

### Recommendation

Further research is needed before a fundamental statement can be made about the studied subject. Future studies should also focus on technological innovations, especially those related to improvements in ultrasound. Standardized detection of suitable veins based on its diameter and the size of the intravenous catheter by ultrasound, can guide decision-making according to peripheral intravenous cannulation. Cannulation success must be of priority in this. Though, consideration of a matching venous diameter to the inserted peripheral intravenous catheter should be part of usual care in vascular access management. For the insertion of a larger sized catheter, a larger sized vein should be selected, logically. To add on this, the current study results should be evaluated prospectively with proper measurements and all attempts recorded. Additionally, awareness should be created for the risk for failed cannulation on the first attempt in patients with an increased risk on the A-DIVA scale, particularly in patients with a venous diameter less than 3 millimeters.

### Limitations

The diameter of the target vein was measured by placing a ruler on the extremity. This way of determining venous size could have resulted in measurement errors. The use of ultrasound will likely result in more accurate outcomes. Nonetheless, the chosen method is easier to apply due to the simple fact that the use of ultrasound is not accessible for every healthcare provider. Furthermore, the result of this study was based on a previous created dataset, which was assessed for the current study [4]. With this design, there is an increased risk for selection bias. To add on this, researchers have limited control over the outcome measurements or exposures in retrospective research. In a retrospective cohort, researchers use preexisting data to identify participants with regard to outcome status to assess the incidence, not just the prevalence, of the outcome of interest [18, 19]. Despite, the current study was based on an existing database with successful cannulation on the first attempt as outcome of interest. Furthermore, venous size is very dependent on environmental factors as ambient temperature, the use of warming elements, clothing, anxiety, vascular diseases or the use of premedication. The stated venous measurements reflects the venous size at the moment of measurement before cannulation, and not the maximum venous size. To add on this, tourniquet placement, tapping over the vein and the use of vasodilator drugs can positively affect venous size and should be taken in consideration in future studies. Further research should be performed on this topic. The employability and usability of calculating a patients CVR should be investigated in a prospective study, especially in those with smaller target veins or those with an indication for a larger sized catheter.

## Conclusion

This first introduction of the CVR in relation to cannulation success should be further investigated. Measuring the venous diameter or detection of a vein with a specific diameter prior to cannulation can possibly increase cannulation success. This is because in this way a suitable vein will be selected based on the size of the chosen catheter, in which the size of the catheter is determined on the indication for the intravenous treatment. Finally, calculation of a patients CVR based on the chosen intravenous catheter can be of added value in first attempt cannulation success, particularly in those patients with a difficult intravenous access or a high score on the A-DIVA scale. An optimal CVR, which is smaller than 0.41, can be achieved by selecting larger sized veins or smaller sized catheters. Ultrasound should be used to select and measure suitable veins on the upper extremity.

## Supporting information

S1 Dataset (XLSX)

S1 File (PDF)
