# Supplementary material for: The impact of the catheter to vein ratio on peripheral intravenous cannulation success, a post-hoc analyses
Source: PLoS One. 2021 May 24;16(5):e0252166. doi: 10.1371/journal.pone.0252166 (PMC8143382; doi:10.1371/journal.pone.0252166)
Supplement: S1 File — (PDF) [file pone.0252166.s002.pdf]

A-DIVA study | Phase 2 | Observation and registration form

Patient's identification

Demographic data

Sex ☐ Male ☐ Female
Age __________ years
Length __________ centimeters
Weight __________ kilograms
ASA class. ☐ 1 ☐ 2 ☐ 3 ☐ 4 ☐ 5
Skin color ☐ Asian ☐ Caucasian ☐ Hispanic / Arabic ☐ Negroid
Patients dominant side
☐ Left ☐ Right
Know history of difficult IV access
☐ Yes ☐ No

NIVAS Grade

Number of suitable veins for cannulation after applying a tourniquet*
☐ 1 ☐ 2 ☐ 3
☐ 4 ☐ 5 ☐ 6 or more

** Veins are suitable for cannulation if the (stewed) vein can by identified by palpating and/or visualizing the extremity and seems fit for cannulation*

Admission to / specialism

Days in hospital __________ days
☐ Anesthesia / OR ☐ Cardiology
☐ Cardio-thoracic ☐ Intensive Care
☐ Emergency dep. ☐ GI surgery
☐ Gynecology ☐ Obstetrics
☐ Plastic surgery ☐ Urology
☐ Neurology ☐ Neurosurgery
☐ Pulmonary ☐ ENT surgery
☐ Eye surgery ☐ Dialyses dep.
☐ Vascular surgery ☐ Other:
____

Indication for IV access

☐ Pre-operative
☐ Antibiotic therapy
☐ Fluid administration
☐ Chemotherapy
☐ Administration of medication

Social behavior

Smoking ☐ No ☐ < 5/day ☐ 5-15/day ☐ > 15/day
Drinking ☐ No ☐ < 5/day ☐ 5-10 /day ☐ > 10/day
Drug abuse ☐ No ☐ Yes: ☐ Smoking ☐ IV drugs

Medical history

Sickle cell disease
☐ Yes ☐ No
Pregnant ☐ No
☐ Yes: __________ weeks of gestation
Cardiac disease ☐ No
☐ Yes, NYHA scale __________
Other: ________________________
Pulmonary disease ☐ No
☐ Yes, COPD Gold __________
Other: ________________________
Chemotherapy ☐ No
☐ Yes, recently ☐ < 1 year ago
☐ 1-5 years ago ☐ > 5 years ago
Diabetes Mellitus ☐ No
☐ Yes, type 1 ☐ Yes, type 2
History of vascular diseases
☐ Yes ☐ No
Dehydrated
☐ Yes ☐ No
☐ Other: ________________________
☐ Other: ________________________
☐ Other: ________________________

Expectation

Do you expect a difficult intravenous access or a failed first attempt of inserting an intravenous catheter in this patient?
☐ Yes ☐ No

© 2016

Attempt 1

Extremity

☐ Left ☐ Right

Position on the extremity

☐ Dorsum hand ☐ Forearm
☐ Elbow crease ☐ Upper arm

Cannulated vein

☐ Cephalic ☐ Basilic
☐ Median ☐ Median cubital

Vein identifiable by palpation?

☐ Yes ☐ No

Vein identifiable by visualization?

☐ Yes ☐ No

Diameter of the cannulated vein

__________ millimeters

Inserted catheter size

☐ 14 gauge ☐ 16 gauge
☐ 17 gauge ☐ 18 gauge
☐ 20 gauge ☐ 22 gauge

Pain score (NRM) upon cannulation

__________ (0 to 10)

Practitioner

☐ Physician ☐ Nurse, specify:
☐ Anesthetist ☐ Emergency dep.
☐ Intensive Care ☐ Physician
☐ Ward (general) ☐ Other:

____

Time needed: __________ minutes

Cannulation successful

☐ Yes ☐ No

Attempt 2

Extremity

☐ Left ☐ Right

Position on the extremity

☐ Dorsum hand ☐ Forearm
☐ Elbow crease ☐ Upper arm

Cannulated vein

☐ Cephalic ☐ Basilic
☐ Median ☐ Median cubital

Vein identifiable by palpation?

☐ Yes ☐ No

Vein identifiable by visualization?

☐ Yes ☐ No

Diameter of the cannulated vein

__________ millimeters

Inserted catheter size

☐ 14 gauge ☐ 16 gauge
☐ 17 gauge ☐ 18 gauge
☐ 20 gauge ☐ 22 gauge

Pain score (NRM) upon cannulation

__________ (0 to 10)

Practitioner

☐ Physician ☐ Nurse, specify:
☐ Anesthetist ☐ Emergency dep.
☐ Intensive Care ☐ Physician
☐ Ward (general) ☐ Other:

____

Time needed: __________ minutes

Cannulation successful

☐ Yes ☐ No

Attempt 3

Extremity

☐ Left ☐ Right

Position on the extremity

☐ Dorsum hand ☐ Forearm
☐ Elbow crease ☐ Upper arm

Cannulated vein

☐ Cephalic ☐ Basilic
☐ Median ☐ Median cubital

Vein identifiable by palpation?

☐ Yes ☐ No

Vein identifiable by visualization?

☐ Yes ☐ No

Diameter of the cannulated vein

__________ millimeters

Inserted catheter size

☐ 14 gauge ☐ 16 gauge
☐ 17 gauge ☐ 18 gauge
☐ 20 gauge ☐ 22 gauge

Pain score (NRM) upon cannulation

__________ (0 to 10)

Practitioner

☐ Physician ☐ Nurse, specify:
☐ Anesthetist ☐ Emergency dep.
☐ Intensive Care ☐ Physician
☐ Ward (general) ☐ Other:

____

Time needed: __________ minutes

Cannulation successful

☐ Yes ☐ No

© 2016

A-DIVA study | Phase 2 | Observation and registration form

Attempt 4

Extremity

☐ Left ☐ Right

Position on the extremity

☐ Dorsum hand ☐ Forearm

☐ Elbow crease ☐ Upper arm

Cannulated vein

☐ Cephalic ☐ Basilic

☐ Median ☐ Median cubital

Vein identifiable by palpation?

☐ Yes ☐ No

Vein identifiable by visualization?

☐ Yes ☐ No

Diameter of the cannulated vein

__________ millimeters

Inserted catheter size

☐ 14 gauge ☐ 16 gauge

☐ 17 gauge ☐ 18 gauge

☐ 20 gauge ☐ 22 gauge

Pain score (NRM) upon cannulation

__________ (0 to 10)

Practitioner

☐ Physician ☐ Nurse, specify:

☐ Anesthetist ☐ Emergency dep.

☐ Intensive Care ☐ Physician

☐ Ward (general) ☐ Other:

____

Time needed: __________ minutes

Cannulation successful

☐ Yes ☐ No

Attempt 5

Extremity

☐ Left ☐ Right

Position on the extremity

☐ Dorsum hand ☐ Forearm

☐ Elbow crease ☐ Upper arm

Cannulated vein

☐ Cephalic ☐ Basilic

☐ Median ☐ Median cubital

Vein identifiable by palpation?

☐ Yes ☐ No

Vein identifiable by visualization?

☐ Yes ☐ No

Diameter of the cannulated vein

__________ millimeters

Inserted catheter size

☐ 14 gauge ☐ 16 gauge

☐ 17 gauge ☐ 18 gauge

☐ 20 gauge ☐ 22 gauge

Pain score (NRM) upon cannulation

__________ (0 to 10)

Practitioner

☐ Physician ☐ Nurse, specify:

☐ Anesthetist ☐ Emergency dep.

☐ Intensive Care ☐ Physician

☐ Ward (general) ☐ Other:

____

Time needed: __________ minutes

Cannulation successful

☐ Yes ☐ No

Other attempts

> 5 attempts ☐ No

☐ Yes: __________ attempts

After > 5 attempts, other techniques applied ☐ No

☐ Ultrasound ☐ VeinViewer®

☐ Central venous cannulation

☐ Other: ________________________

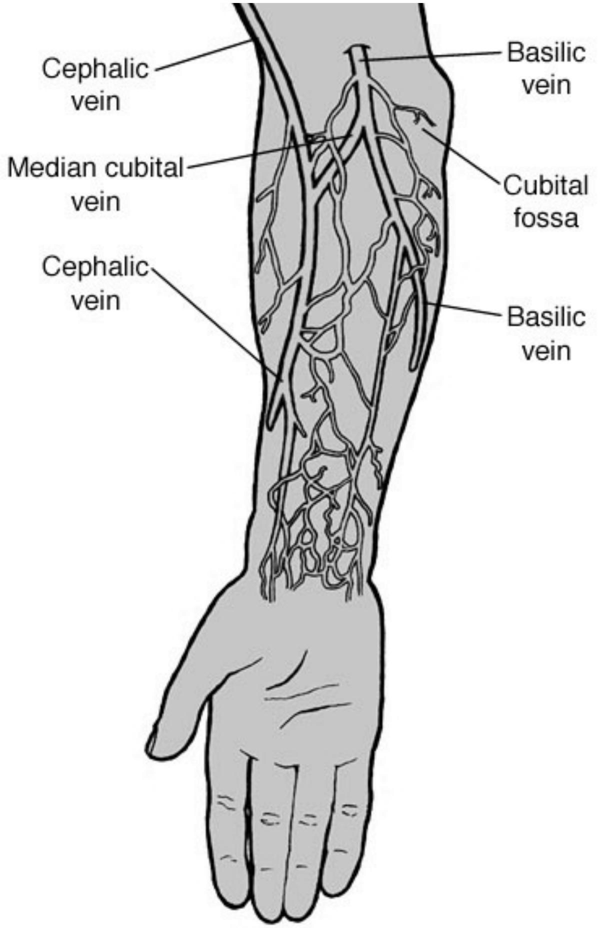

F.H.J. van Loon, MSc; H. de Boer, MD; K.B. Botelho, RN, BSN; L.A.P.M. Puijn RN; R.A. Bouwman MD

© 2016
